# Supplementary material for: k-mer manifold approximation and projection for visualizing DNA sequences
Source: Genome Res. 2025 May;35(5):1234–46. doi: 10.1101/gr.279458.124 (PMC12047656; doi:10.1101/gr.279458.124)
Supplement: Supplement 8 [file Supplemental_Data_1.zip › Supplemental_Data_1/NKX3-1_TCCCTC20NCG_AD_3/NKX3-1_TCCCTC20NCG_AD_3_MDS.pdf]

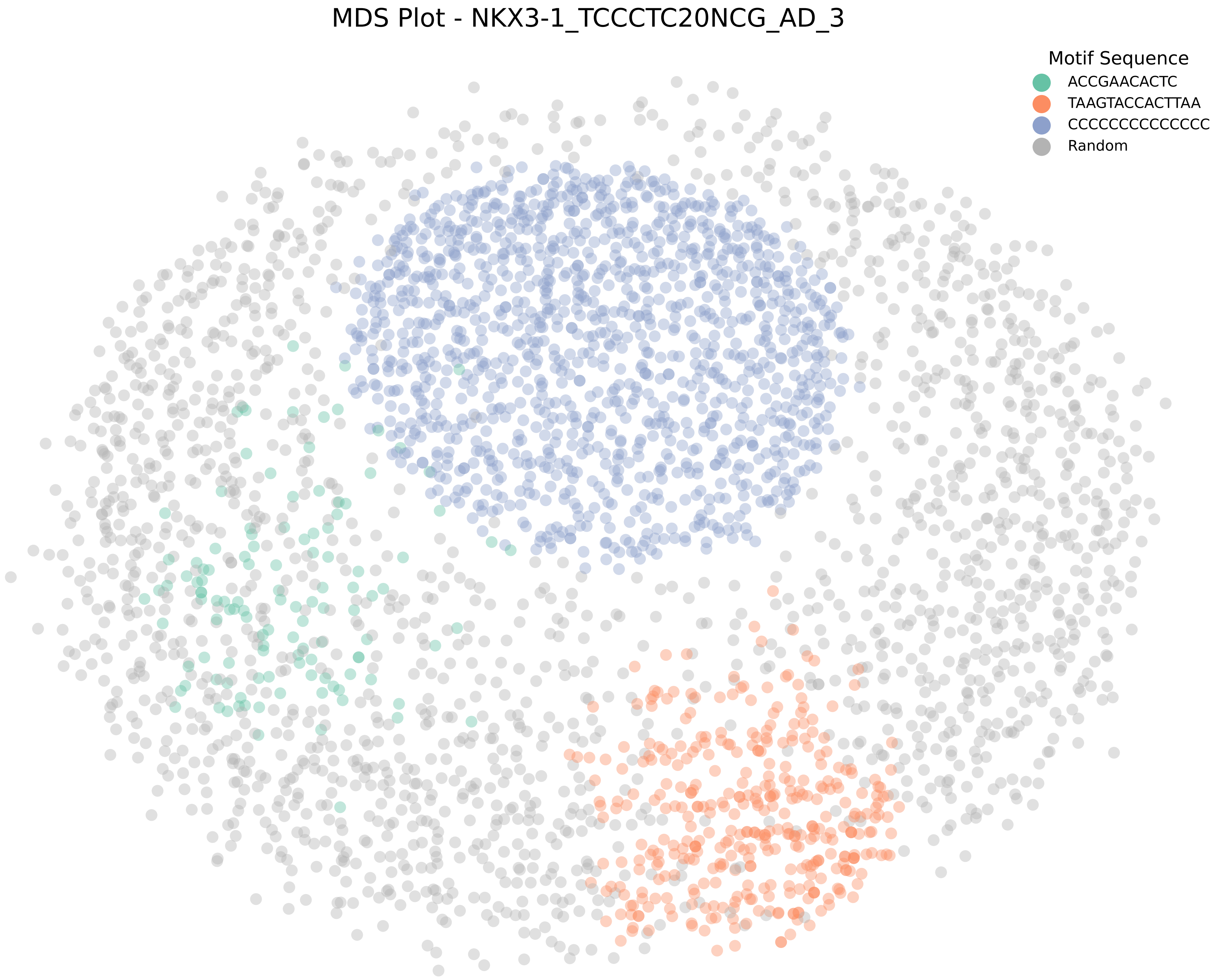
MDS Plot - NKX3-1_TCCCTC20NCG_AD_3
Motif Sequence
ACCGAACACTC
TAAGTACCACTTAA
CCCCCCCCCCCCCC
Random
